# Cytoplasmic proliferating cell nuclear antigen connects glycolysis and cell survival in acute myeloid leukemia

**DOI:** 10.1038/srep35561

**Published:** 2016-10-19

**Authors:** Delphine Ohayon, Alessia De Chiara, Nicolas Chapuis, Céline Candalh, Julie Mocek, Jean-Antoine Ribeil, Lamya Haddaoui, Norbert Ifrah, Olivier Hermine, Frédéric Bouillaud, Philippe Frachet, Didier Bouscary, Véronique Witko-Sarsat

**Affiliations:** 1 INSERM U1016, Institut Cochin, Paris, France; 2 Université Paris Descartes, Faculté de Médecine Sorbonne Paris Cité, Paris, France; 3 CNRS UMR 8104, Paris, France; 4 Center of Excellence, Labex Inflamex, France; 5 Hematology Department, Cochin Hospital, Assistance publique-Hôpitaux de Paris (APHP), Paris, France; 6 FILO: French Innovative Leukemia Organization (GOELAMS), CHU Bretonneau, TOURS France; 7 Biotherapy Department, Necker Hospital, Paris, France; 8 Hematology Department CHU & UMR INSERM U892/CNRS6299, Université d’Angers, France; 9 Hematology Department, Necker Hospital Assistance publique-Hôpitaux de Paris (APHP), France; 10 INSERM UMR1163, CNRS ERL 8254, Institut Imagine, Paris, France; 11 Institut de Biologie Structurale, Centre Etude Atomique, Grenoble, France; 12 Université Grenoble Alpes, CNRS, UMR 5075, Grenoble, France

## Abstract

Cytosolic proliferating cell nuclear antigen (PCNA), a scaffolding protein involved in DNA replication, has been described as a key element in survival of mature neutrophil granulocytes, which are non-proliferating cells. Herein, we demonstrated an active export of PCNA involved in cell survival and chemotherapy resistance. Notably, daunorubicin-resistant HL-60 cells (HL-60R) have a prominent cytosolic PCNA localization due to increased nuclear export compared to daunorubicin-sensitive HL-60 cells (HL-60S). By interacting with nicotinamide phosphoribosyltransferase (NAMPT), a protein involved in NAD biosynthesis, PCNA coordinates glycolysis and survival, especially in HL-60R cells. These cells showed a dramatic increase in intracellular NAD+ concentration as well as glycolysis including increased expression and activity of hexokinase 1 and increased lactate production. Furthermore, this functional activity of cytoplasmic PCNA was also demonstrated in patients with acute myeloid leukemia (AML). Our data uncover a novel pathway of nuclear export of PCNA that drives cell survival by increasing metabolism flux.

Proliferating cell nuclear antigen (PCNA) is an ancestral molecule with a master role in keeping the organism alive through its pivotal function in DNA replication and repair^1^. PCNA is the only protein that acts as a sliding clamp, which is a cofactor of DNA polymerase that encircles DNA and prevents the enzyme from dissociating from the template DNA during synthesis^2^. PCNA has no intrinsic enzymatic activity and its main function is to build a protein scaffold through the binding and functional coordination of its different partners^3^. PCNA interacts with numerous proteins and it is believed that the switching of PCNA partners may be triggered by affinity-driven competition or by specific post-translational modifications of PCNA. Nuclear PCNA has a well-documented role in cancer and through its ability to control the replication fork is central in determining both tumor progression as well as anticancer treatment outcomes^4^. As a corollary, nuclear PCNA has been proposed as a potential target for inhibiting cell proliferation in cancer.

However, the importance of PCNA in controlling cell fate extends far beyond DNA-related processes since we previously identified an unexpected anti-apoptotic function for PCNA in neutrophils, which are highly differentiated cells that are devoid of proliferative capacities^5^. Notably, neutrophils have specific pathways to control their balance between death and survival^6^. For instance, even if neutrophils cannot proliferate, their survival is controlled by cell cycle proteins such as survivin^7^ and cyclin-dependent kinases^6^, the inhibition of which by roscovitine can trigger apoptosis^8^. Remarkably, within neutrophils, PCNA is exclusively localized in the cytoplasm and is associated with procaspases, which prevents their activation^9^. However, under inflammatory conditions, inducible proteins such as the cyclin-dependent kinase inhibitor p21/waf1 can regulate the PCNA scaffold^10^. PCNA could also regulate neutrophil survival during infections as it has been suggested during *Staphylococcus aureus* infection^11^. These studies are in agreement with the notion that the cytosolic scaffold of PCNA is strictly adjusted to the physiology of neutrophils to promote survival^12^,^13^. It is worth noting in this regard that the relocation of PCNA from the nucleus into the cytoplasm occurs at the end of granulocytic differentiation and involves chromosome region maintenance 1 (CRM1)-dependent nuclear export characterized by the presence of a nuclear export sequence (NES) which is surface-exposed only when PCNA is monomeric^14^,^15^. During normal myelopoiesis leading to granular differentiation, the exclusive cytoplasmic localization of PCNA, observed only in mature neutrophils, is both a trademark of terminal differentiation and a key component of neutrophil survival^5^. Conversely, we have observed that normal hematopoietic progenitor cells at the promyelocytic stage harbor mostly nuclear PCNA as these cells retain a proliferative capacity. It is not known whether the export of PCNA could be dysregulated in myeloid precursors and whether this could impact on the survival of the cells. It is generally accepted that leukemic cells invariably possess abnormalities in one or more cell death pathways, which confer a survival advantage of these cells over their normal counterparts^16^. Furthermore, abnormalities in the apoptotic response also play a role in the development of drug resistance by leukemic cells^17^. In the current study, we first examine PCNA localization in the HL-60 promyelocytic cell line and whether PCNA could participate to the survival mechanisms involved in resistance to chemotherapy. We next investigate PCNA localization in blasts from patients with acute myeloid leukemia (AML).

## Results

### Increased cytoplasmic PCNA is observed in HL-60R resistance to chemotherapy and is associated with a survival advantage in daunorubicin-resistant HL-60 cells

**Figure 1:**
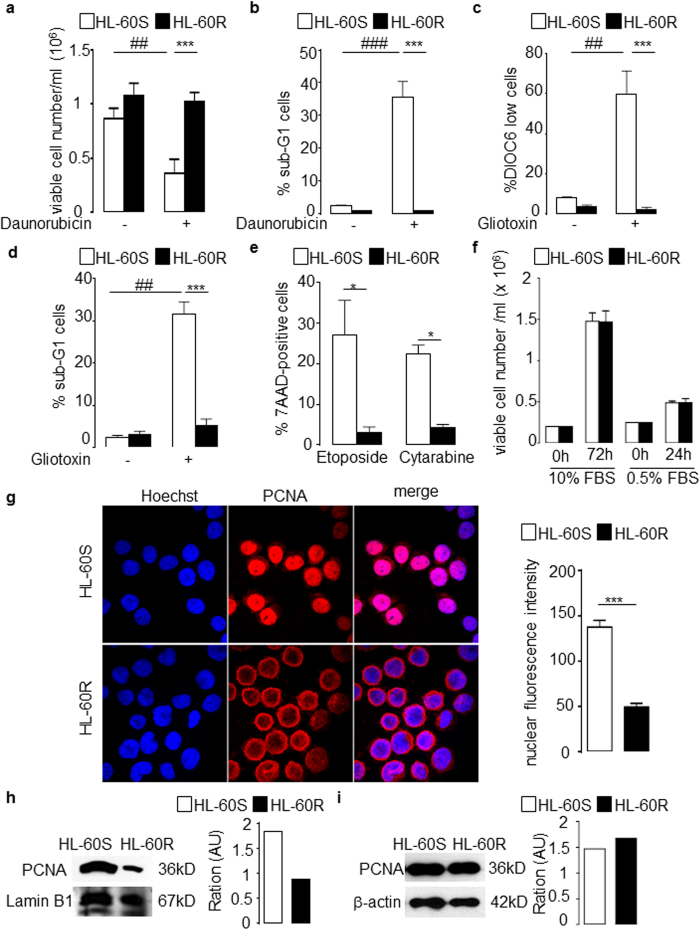
Functional characterization and cytoplasmic PCNA localization in daunorubicin- sensitive (HL-60S) and – resistant (HL-60R) cells. (**a,b**) HL-60 cells were treated with or without daunorubicin for 16 h. Data are mean ± SEM of six independent experiments performed in duplicates. (**a**) Viable cells were counted using flow cytometry. (**b**) Percentage of cells in the sub-G1 phase after propidium iodide labeling. (**c,d**) HL-60 cell lines were incubated with or without gliotoxin for 16 h to induce apoptosis. (**c**) Percentages of cells after DIOC6 labeling. Values are means ± SEM of four independent experiments. (**d**) Percentages of cells in sub-G1 phase showing DNA fragmentation after propidium iodide labeling. Values are means ± SEM of five independent experiments. (**e**) Effect of etoposide and cytarabine treatment in HL-60 cell lines for 16 h. Percentages of 7AAD + cells determined by flow cytometry. Values are means ± SEM of three independent experiments performed in duplicates. (**f**) Numbers of HL-60 cell lines counted after Trypan blue exclusion, where 1 × 10^6^ cells were cultivated in 5 ml of RPMI for 72 h in the presence of 10% FBS or 24 h in the presence of 0.5% FBS. Values are means ± SEM of six (at 72 h) and five (at 24 h) independent experiments. (**g**) Immunofluorescence analysis of PCNA localization by confocal microscopy (nuclei are visualized using Hoechst). This representative experiment was performed more than 10 times with identical results. Right panel represents quantification of nuclear fluorescence intensity by Image J. Data are means ± SEM of 15 values obtained after quantification of three independent experiments. (**h,i**) Western blot analysis of PCNA expression in HL-60 cell lines (**h**) in nuclear fractions using 20 μg protein/lane. Results were quantified by densitometry and normalized to lamin B1 loading control. The same experiments were performed with the whole lyzate using 10 μg protein/lane. Results were quantified by densitometry and normalized to β-actin loading control. For (**a–f**) multicomparison ANOVA test (indicated by*) was used to evaluate the difference between HL-60S and HL-60R and Student’s *t* test (indicated by #) was used to evaluate the effect of the drug on either HL-60S or HL-60R, *p < 0.05, **p < 0.01, ***p < 0.001.

We examined the relationship between PCNA subcellular localization and daunorubicin resistance. A significant increase in the viability of daunorubicin-resistant HL-60 (HL-60R) cells was observed compared to daunorubicin-sensitive HL-60 (HL-60S) cells (Fig. 1a). In contrast to HL-60S, no increase in apoptosis was observed in HL-60R cells treated by daunorubicin, as measured by the percentage of cells in the sub-G1 phase (Fig. 1b). Gliotoxin induced significant apoptosis in HL-60S but not HL-60R cells, as assessed by both mitochondria depolarization (Fig. 1c) and the percentage of cells in sub-G1 phase (Fig. 1d). HL-60R cells were also resistant to cytarabine and etoposide, two drugs commonly used to treat AML (Fig. 1e). No difference in proliferation was observed between both cell types when cultured in the presence of 10% or 0.5% FBS (Fig. 1f) demonstrating that HL-60R cells had acquired a multidrug resistant phenotype without affecting proliferation. As cytosolic PCNA can potentiate survival in a cell cycle-independent manner in neutrophils^9^, PCNA subcellular localization was examined by immunofluorescence in both HL-60S and HL-60R cells. There was a significant increase in cytoplasmic PCNA in HL-60R compared to HL-60S cells (Fig. 1g, left panel). Conversely, PCNA nuclear fluorescence intensity was significantly decreased in HL-60R cells compared to HL-60S (Fig. 1g, right panel). This was confirmed by Western blot analysis of the nuclear fractions of both HL-60S and HL-60R (Fig. 1h). Of note, the total amount of PCNA within the whole cell lysate was similar in both cell lines (Fig. 1i). Notably, PCNA subcellular localization was also examined in K562 cells which are cells of erythroleukemia type derived from a patient with chronic myeloid leukemia and are positive for bcr:abl fusion gene. Like in HL-60R cells, K562 resistant to doxorubicin or to the tyrosine kinase inhibitor imatinib, had a prominent cytosolic localization of PCNA whereas the control K562 cells had a nuclear localization (Supplementary Figure S1).

### Cytoplasmic localization of PCNA in HL-60R cells is dependent on CRM1

**Figure 2:**
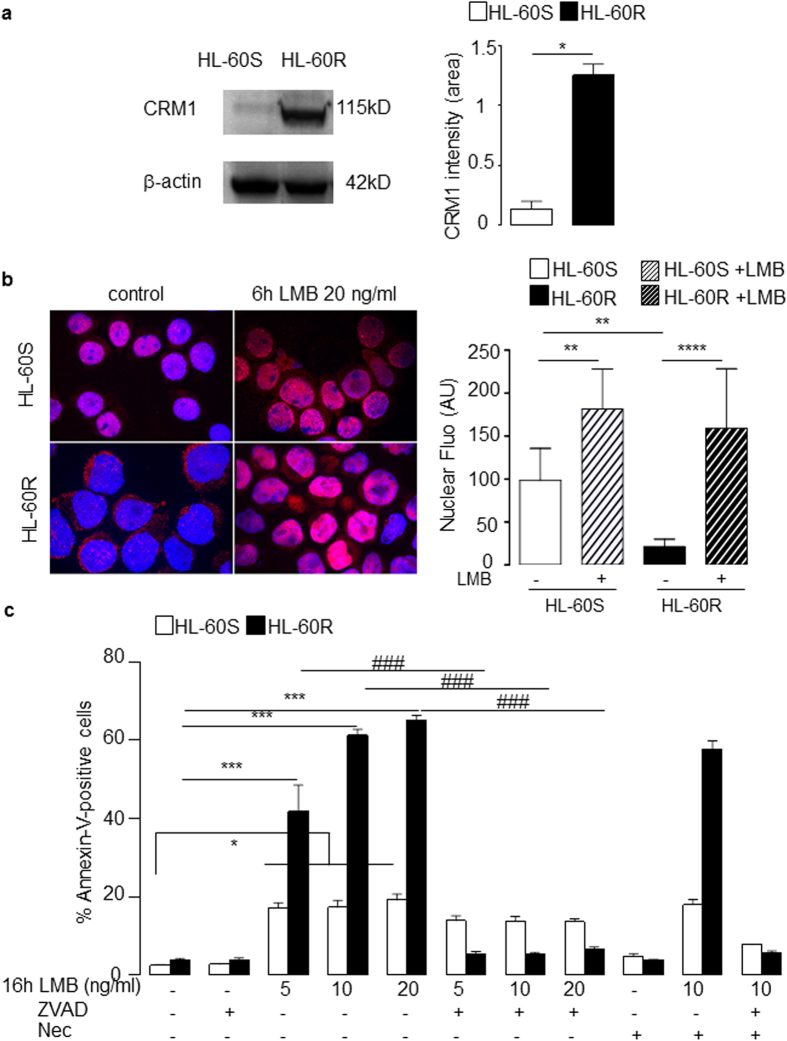
The nuclear export of PCNA is increased in HL-60R cells and influenced their viability. (**a**) Western blot analysis of CRM1 expression in whole cell lysates from HL-60S and HL-60R cells using anti-CRM1 rabbit polyclonal Ab (2 × 10^6^ cells/lane) and β-actin for the loading control. This experiment has been performed four times with identical results. Results were quantified by densitometry and normalized to *β*-actin loading control. The data are mean ± SEM of four independent experiments, *p < 0.05 (Student’s *t* test). (**b**) Effect of leptomycin B (LMB) on PCNA subcellular localization assessed by indirect immunofluorescence using anti-PCNA Ab5 rabbit polyclonal Ab. HL-60S and HL-60R cells were treated for 6 h with 20 ng/ml LMB. This experiment was performed four times with identical results. Right panel represents quantification of nuclear fluorescence intensity by Image J 1.48v soft©ware. Data are means ± SEM of at least 15 values obtained after quantification of four independent experiments. (**c**) Effect of LMB on the apoptosis of HL-60S and HL-60R. HL-60 cells cultured for 16 h at 37 °C in the absence or presence of LMB (5 or 10 or 20 ng/ml) or the pan caspase inhibitor Z-VAD(OMe)-FMK (50 μM) or the necroptosis inhibitor necrostatin-1 (Nec, 50 μM). The percentages of apoptotic cells were measured by annexin-V^+^ and 7-AAD^−^ using flow cytometry. The data are the mean ± SEM of six independent experiments, ***p < 0.001 (Student’s *t* test) for comparaison between HL-60R and HL-60S and ^###^p < 0.001 (Student’s *t* test) for comparaison between untreated and Z-VAD(OMe)-FMK-treated HL-60R cells.

Western blot analysis revealed that CRM1 expression was dramatically increased in HL-60R compared to HL-60S cells (Fig. 2a). Following a 6h treatment with leptomycin B (LMB), a specific inhibitor of CRM1-dependent nuclear export, PCNA nuclear accumulation in HL-60R cells was observed by immunofluorescence at a higher level than HL-60S cells (Fig. 2b). Quantification of the nuclear fluorescence intensities first showed a statistically significant lower nuclear fluorescence of PCNA in the HL-60R compared to HL-60S in the absence of LMB as already shown in Fig. 1g. The effect of LMB could be observed in both HL-60S and HL-60R suggesting that the cytosolic PCNA localization involved a CRM1-dependent export in both cell lines. However the percentage of increase of nuclear fluorescence following LMB treatment was higher in HL-60R compared with HL-60S with a mean ± SEM of 90.7 ± 3.1% versus 45.1 ± 11.8%, respectively. After a 16 h overnight LMB treatment, HL-60S apoptosis was induced by LMB at 5, 10 and 20 ng/ml. However, the pro-apoptotic effect of LMB was dramatically higher in HL-60R cells and was reversed by the pan-caspase inhibitor Z-VAD(OMe)-FMK but not by necrostatin, a necroptosis inhibitor (Fig. 2c). Western blot analysis of procaspase 3 (Supplementary Figure S2a) and procaspase 9 (Supplementary Figure S2b) showed an increased expression at the basal level in HL60R as compared with HL60S. In addition, the activated caspase-3 band at 17 kDa could be clearly detected in LMB-treated HL60R cells and was sensitive to ZVAD treament, whereas no activated caspase-3 band could be observed in LMB-treated HL60S cells. Likewise, the band of activated caspase-9 could be detected at 37 kDa only in LMB-treated HL60R and not in HL60S cells. Pertinently, the 37 kDa activation band of caspase 9 was sensitive to ZVAD treatment in HL60R cells. Of note, no difference in procaspase 8 expression between HL60S and HL60R was observed (Supplementary Figure S2c). Taken together, our results revealed that high expression of cytosolic PCNA in HL-60R cells was associated with an increase in CRM1-dependent export, the inhibition of which triggered a caspase-dependent apoptosis response only in HL60R cells.

### Suppression of cytosolic PCNA function or expression promotes apoptosis in HL-60R cells

**Figure 3:**
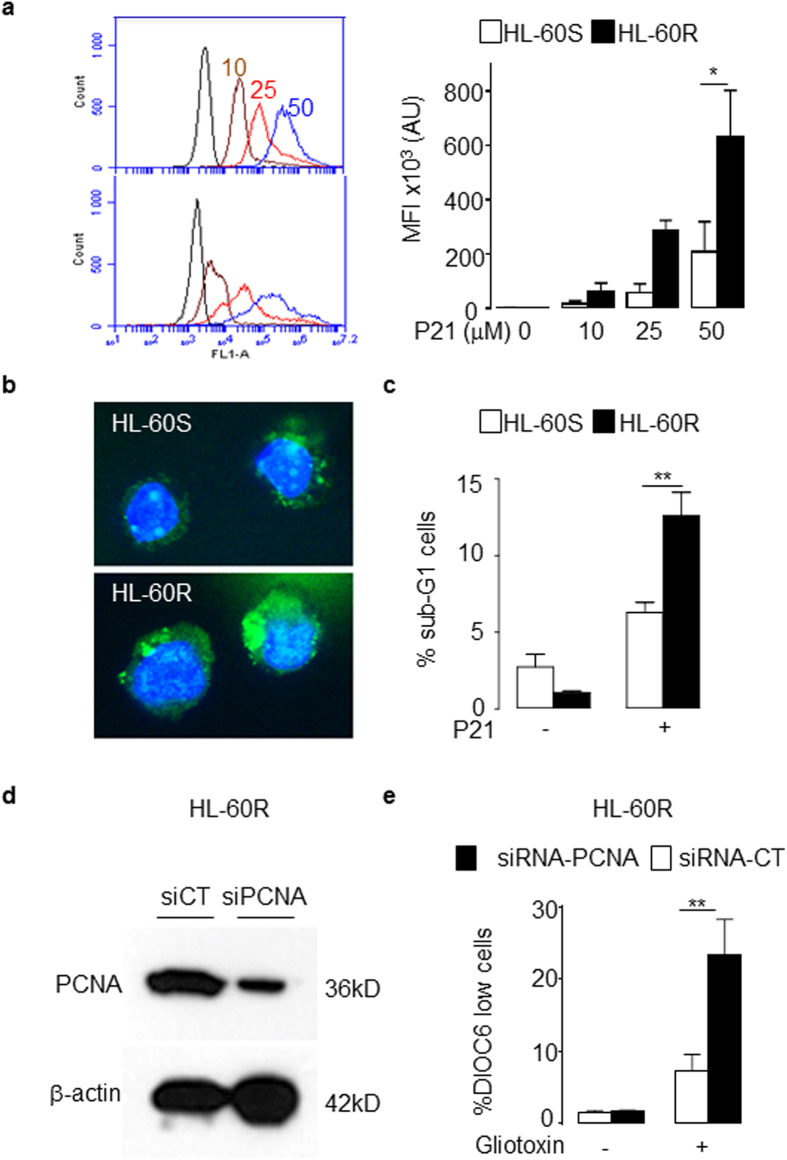
PCNA siRNA and p21-peptide-YIRS decreased the viability in HL-60R cells. (**a**, left panel) Representative flow cytometry analysis plots of HL-60S and HL-60R treated with FITC-p21-peptide-YIRS. HL-60 cells exposed to increasing concentrations of the FITC-p21-peptide-YIRS (0, 10 (braun), 25 (red) and 50 (blue) μM) for 30 min. Experiment was performed four times with identical results. (**a**, right panel) Quantification of mean fluorescence intensity (MFI) of HL-60S and HL-60R treated with FITC-p21-peptide-YIRS. The data are mean ± SEM of four independent experiments, *p < 0.05 (ANOVA test). (**b**) Confocal microscopy analysis used to determine the localization of the FITC-p21-peptide-YIRS (1 h, 25 μM) in HL-60S and HL-60R. The FITC-p21-peptide-YIRS is visualized by green fluorescence and nucleus by Hoechst staining in blue. This experiment was performed three times with identical results. (**c**) Effect of p21-peptide-YIRS on DNA fragmentation. HL-60 cells were cultured for 16 h at 37 °C with or without 50 μM of p21-peptide-YIRS. Apoptosis was assessed by the percentage of cells in sub-G1 after propidium-iodide staining. The data are mean ± SEM of four independent experiments. **p < 0.01 (ANOVA test) (**d**) Representative Western blot analysis of HL-60R cells (50 μg/lane) showing decreased PCNA expression after PCNA siRNA treatment compared with control siRNA. Experiment was performed four times with identical results. (**e**) Apoptosis was induced by gliotoxin (1 μg/ml) for 4 h and mitochondria depolarization after DIOC6 labeling assessed. The data are mean ± SEM of four independent experiments, **p < 0.01 (ANOVA test).

The p21-peptide-YIRS which binds to the interdomain connecting loop of PCNA^18^ was used to inhibit PCNA function in our current experiments. Flow cytometry analysis demonstrated that FITC-p21-peptide-YIRS penetrated cells in a dose-dependent manner (Fig. 3a). HL-60R cells displayed an increased FITC-p21-peptide-YIRS MFI compared with HL-60S cells due to an increased abundance of cytosolic PCNA in HL-60R cells (Fig. 3a, right panel). Furthermore, the FITC-p21-peptide-YIRS was found to be localized prominently within the cytosol (Fig. 3b). Furthermore, p21-peptide-YIRS induced DNA fragmentation to a greater extent in HL-60R compared with HL-60S cells (Fig. 3c). Next, siRNA was used to specifically down-regulate the expression of PCNA in HL-60R cells as validated by Western blot analysis (Fig. 3d). Treatment with PCNA siRNA, but not control siRNA, significantly sensitized HL-60R cells to gliotoxin-induced apoptosis (Fig. 3e).

### Direct interaction between PCNA and NAMPT in the cytoplasm modulates the intracellular NAD+ level and glycolysis in HL-60R cells

**Figure 4:**
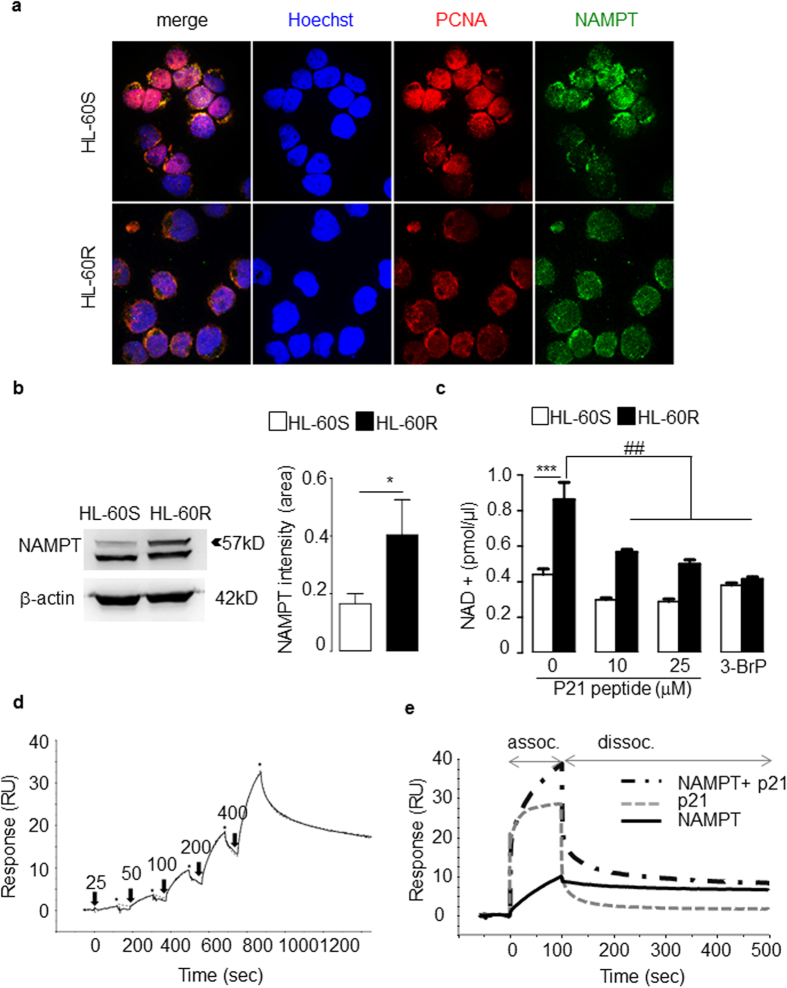
Analysis of NAMPT expression in HL-60 cells. (**a**) Co-localization experiments of NAMPT (green staining) and PCNA (red staining) in HL-60S and HL-60R using confocal immunofluorescence analysis. Hoechst was used for nuclear staining. This experiment has been performed three times with identical results. (**b**) Western-blot analysis of cytosolic NAMPT expression in HL-60 cell lines. Cytosols were obtained by nitrogen cavitation and 50 μg of protein loaded per lane. Results were quantified by densitometry of the top band and normalized to β-actin loading control. The data are mean ± SEM of five independent experiments, *p < 0.05 (Student’s t test). (**c**) Effect of the p21-peptide-YIRS on NAD concentrations in HL-60S and HL-60R. Measurement of NAD concentration after 6 h treatment with the p21-peptide-YIRS or with 3-bromopyruvate (3-BrP) used as a glycolysis inhibitor. The data are mean ± SEM of four independent experiments performed in duplicates. Multicomparison ANOVA test (indicated by*) was used to evaluate the difference between HL-60S and HL-60R and Student’s t test (indicated by #) was used to evaluate the effect of the drug on either HL-60S or HL-60R, *p < 0.05, **p < 0.01, ***p < 0.001. (**d**) Measurement of the interaction between PCNA and NAMPT using surface plasmon resonnance SPR analysis. A typical sensorgrams showing injection of NAMPT at the indicated concentrations over PCNA covalently immobilized on a CM5 sensor chip is shown (RU, response units). Arrows and stars indicate the injection start and end points for each concentration. A fit representing a typical experiment performed three times is shown, and was obtained by global fitting of the data using the 2-state model. The kinetic parameters of the interaction determined by analyzing response at varying NAMPT concentrations are listed in Supplementary Table 2. (**e**) P21-peptide binding to PCNA does not interfere with NAMPT–PCNA association. An overlay of the sensorgrams obtained with NAMPT, P21-peptide and a mixture of both on PCNA is showed (RU, response units). Binding assay was performed on a Biacore T200 system as described in Methods. This representative experiment has been performed twice giving identical results.

As PCNA functions are dependent on its partners, the PCNA interactome was analyzed in both HL-60R and HL-60S cells. PCNA was found to be associated with enzymes involved in metabolism (Supplementary Table 1). Interestingly, several PCNA-associated-proteins, involved in glycolytic or nuclear export pathways, showed differences between HL-60S and HL-60R. One of the PCNA partners preferentially identified in the HL-60R interactome was nicotinamide phosphoribosyltransferase (NAMPT), initially named pre-B-cell colony enhancing factor (PBEF) or visfatin^19^. NAMPT catalyzes the first step in the biosynthesis of nicotinamide adenine dinucleotide (NAD) from nicotinamide (NAM) and controls glycolysis^20^. Using confocal microscopy, double immunolabeling of NAMPT and PCNA showed clear evidence that both proteins colocalized in HL-60R cell cytosol (Fig. 4a). Moreover, Western blot analysis showed an increased NAMPT content in HL-60R compared to HL-60S cells (Fig. 4b). NAMPT expression appeared as a doublet at 52 and 57 kDa, presumably due to autophosphorylation^21^.

As NAMPT is involved in NAD salvage pathway tightly associated with glycolysis^22^, we hypothesized that its increased expression in HL-60R cells may be associated with increased NAD concentrations. Accordingly, the NAD+ levels were significantly higher in HL-60R compared with HL-60S cells (Fig. 4c). Furthermore, the NAD+ concentrations were modulated by 3-bromopyruvate (3-BrP), an inhibitor of the glycolytic pathway (Fig. 4c). The p21-peptide-YIRS induced a dramatic decrease in NAD+ concentrations in HL-60R cells with only a moderate effect on HL-60S (Fig. 4c). This result suggested that intracellular NAD+ concentrations are modulated by the PCNA scaffold. Using SPR with recombinant PCNA and purified NAMPT, a direct interaction was observed with an apparent KD (*K*_D_ = 1.8 × 10–7M) value in the submicromolar range (Fig. 4d). Proper kinetic analysis of this interaction required the use of a two-state reaction binding model, which takes into account complex conformational changes^23^. The two groups of kinetic constants and the apparent affinity constant (*K*_D_) are listed in Supplementary Table 2. The fact that the experimental binding data can be properly fitted using this model suggested that NAMPT and PCNA form a stable complex that may involve a conformational change. The ability of the p21-peptide, known to bind the interdomain connecting loop of PCNA, to interfere with the NAMPT-PCNA interaction was examined. When the NAMPT-p21 mixture (Fig. 4e) was injected, the association measured was the sum of the NAMPT and the p21 signals obtained separately. This suggested that PCNA-NAMPT association is independent of the interdomain connecting loop or involves two (or more) sites on PCNA that are distinct from the interdomain connecting loop.

### Increased glycolysis in HL-60R cells is affected by the p21 peptide targeting cytosolic PCNA

**Figure 5:**
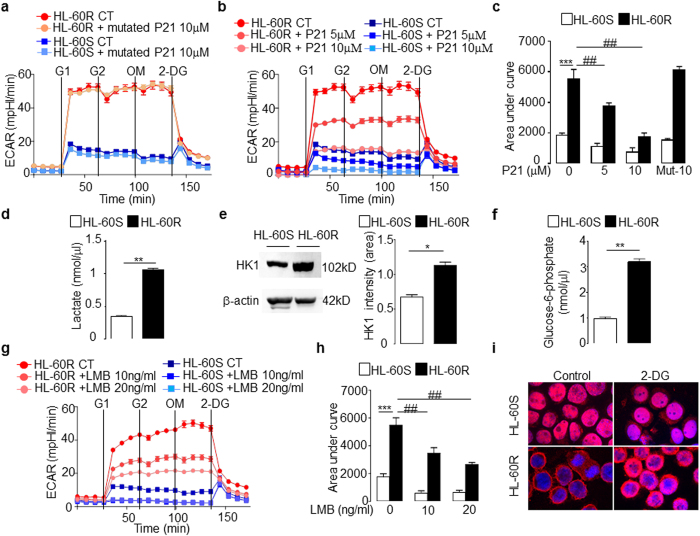
Metabolic characterization of HL-60S and HL-60R cells. (**a–c**) Glycolytic flux measured as extracellular acidification rate (ECAR) of HL-60 cells at baseline and after adding D-glucose at two different concentrations, after oligomycin (OM), and after 2-DG with or without mutated p21-peptide-YIRS (**a**) or p21-peptide-YIRS (**b**). In (**a**,**b**), each panel represents one experiment in which the data plotted on the graph is the mean ± SEM (n = 5). Each experiment has been performed three times with identical results. (**c**) Quantification of the ECAR by measuring the total area under the curve. The data are mean ± SEM of three independent experiments performed in quintuplets. Multicomparison ANOVA test (indicated by*) was used to evaluate the difference between HL-60S and HL-60R and Student’s t test (indicated by #) was used to evaluate the effect of the p21 peptide on either HL-60S or HL-60R, **p < 0.01, ***p < 0.001. (**d**) Measurement of lactate in medium of HL-60S and HL-60R cells. The data are mean ± SEM of four independent experiments performed in duplicates, **p < 0.01 (Student’s t test). (**e**) Western blot analysis of hexokinase 1 in HL-60 cells (50 μg/lane). Quantification by densitometry of the top band is normalized to β-actin loading control. Data are mean ± SEM of three independent experiments, *p < 0.05 (Student’s t test). (**f**) Glucose-6-phosphate concentration in HL-60 cells. The data are mean ± SEM of three independent experiments, **p < 0.01 (Student’s t test). (**g**) Kinetic ECAR response of HL-60 cells after LMB treatment. The panel represents one experiment where the data plotted on the graph is the mean ± SEM of five values. (**h**) Results from (**g**) were quantified by total area under the curve. The data are mean ± SEM of five values. Multicomparison ANOVA test (indicated by*) was used to evaluate the difference between HL-60S and HL-60R and Student’s *t* test (indicated by #) was used to evaluate the effect of the LMB on either HL-60S or HL-60R, **p < 0.01, ***p < 0.001. (**i**) Confocal microscopy analysis of PCNA localization in HL-60 cells and Hoechst for nuclear labeling. Cells were treated for 6 h with 2-DG at 50 mM. Panel i shows a representative experiment of three all yielding the same results.

The glycolysis pathway was next investigated using an extracellular flux analyzer. HL-60R cells showed a significantly higher extracellular acidification rate (ECAR) compared with HL-60S cells. Oligomycin, which blocks oxidative phosphorylation, did not affect the ECAR in either cell type indicating the prominence of the glycolytic pathway in these cells. This was further confirmed by our finding of a significant inhibitory effect of 2-Deoxy-D-glucose (2-DG) on ECAR (Fig. 5a). Again, the p21-peptide-YIRS significantly decreased ECAR in a dose-dependent manner in both HL-60S and HL-60R cells (Fig. 5b) with no effect observed using the mutated p21-peptide-YIRS (Fig. 5a). Notably, the variation in the area under the curve induced by the p21-peptide-YIRS was greater in HL-60R cells compared with HL-60S cells (Fig. 5c). Accordingly, an increased concentration of lactate was observed in the culture media of HL-60R compared to HL-60S (Fig. 5d). Moreover, a significantly increased expression of hexokinase 1, the main enzyme controlling the glycolytic pathway, and glucose-6-phosphate (G6P), its enzymatic product, were detected in HL-60R cells indicating that the initial steps of the glycolytic pathway were stimulated (Fig. 5e,f). However, it should be noted that the oxygen consumption rate (OCR) was low in both the HL-60S and HL-60R cells but still significantly higher in the HL-60R cells (Supplementary Figure S2d). This observation suggested that the large increase in glycolysis in the HL-60R cells is not the result of decreased respiration. Notably, with regard to ECAR and OCR, the HL-60R cells are hypermetabolic and this metabolism is essentially driven by augmented glycolytic activity.

As previously mentioned, the increased glycolysis observed in HL-60R cells was not associated with an increased proliferation rate (Fig. 1f) but was indicative of the so-called Warburg effect, which states that leukemic cells preferentially use an aerobic glycolytic metabolism instead of an oxidative process to promote cancer cell survival. Our data suggest that there is a strong relationship between glycolysis and PCNA-mediated survival. Accordingly, in our current experiments, LMB-treated HL-60R cells displayed a significant and dose-dependent decrease in glycolysis as measured by ECAR (Fig. 5g,h) without affecting cell viability at this time point (Supplementary Figure S2e), again suggesting that PCNA export, by promoting glycolysis, favors cell survival in HL-60R cells. Conversely, inhibition of glycolysis by 2-DG resulted in the accumulation of PCNA within the nucleus in HL-60R cells only, suggesting that the energy generated by the glycolytic pathway was required for export of nuclear proteins including PCNA (Fig. 5i).

### Cytosolic PCNA localization is found in primary leukemic cells from patients with AML

**Figure 6:**
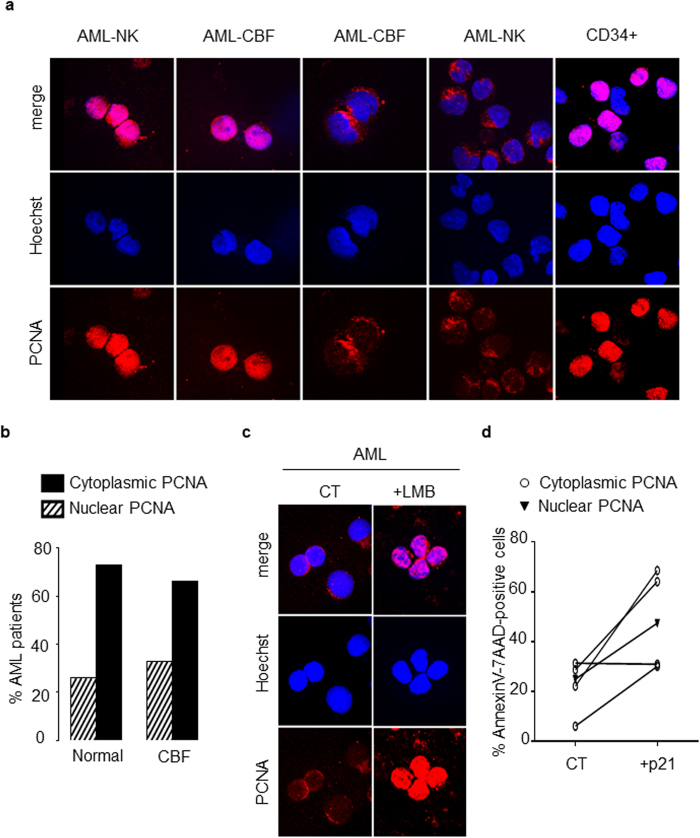
Analysis of PCNA subcellular localization in blast cells from patients with AML. (**a**) Subcellular localization of PCNA in primary leukemic cells from patients with acute myeloid leukemia (AML). Immunofluorescence analysis by confocal microscopy of PCNA localization in blasts of AML patients with NK or CBF and in normal human CD34^+^ HPs using the rabbit polyclonal antibody Ab5 and Hoechst for nuclear labeling. Panel a shows a representative experiment of three, all yielding the same results. (**b**) Distribution of cytoplasmic or nuclear PCNA in blasts from AML patients according to the cytogenetic characterization. PCNA localization was determined in a cohort of 50 AML patients. The histogram depicts the percentages of AML patients showing either cytoplasmic or nuclear PCNA. (**c**) Immunofluorescence analysis by confocal microscopy of PCNA localization (performed as in a) in blasts from one AML patients after LMB treatment. Panel c shows a representative experiment of three yielding the same results. (**d**) Effect of the p21-peptide-YIRS on viability of blasts from 5 AML patients including 4 patients with cytosolic (open circle) and 1 patient with nuclear (dark triangle) PCNA. Blast cells were cultured for 16 h at 37 °C with or without the p21-peptide-YIRS (50 μM) and annexinV-7AAD-positive cells measured by flow cytometry.

PCNA localization was next examined in normal CD34+ HPCs from 3 controls in comparison with primary blasts from 69 AML patients. As we previously described^9^, normal human CD34+ HPs displayed predominantly nuclear PCNA staining (Fig. 6a). PCNA localization was also evaluated using confocal microscopy in leukemic cells analyzed at diagnosis. Two distinct patterns of PCNA localization in blast cells (Fig. 6a) were identified: a nuclear/mixed pattern similar to control CD34+ cells or an exclusively cytosolic pattern. It should be noted that the “nuclear” pattern evident in primary AML blast cells was similar to the pattern observed in HL-60S cells showing both nuclear but also cytosolic PCNA. The cytosolic PCNA pattern was recorded in 46 out the 69 AML patients (66.7%). Among these patients, 50 were issued from the GOELAMS: 26 had normal karyotype AML (NK-AML) and 24 had CBF-AML. As indicated in Fig. 6b, the number of AML patients with cytoplasmic PCNA localization was 19/26 (73.1%) versus 16/24 (66.7%) of patients among NK-AML and CBF-AML cohorts, respectively, suggesting that cytoplasmic PCNA localization was not dependent on the karyotype.

**Figure 7:**
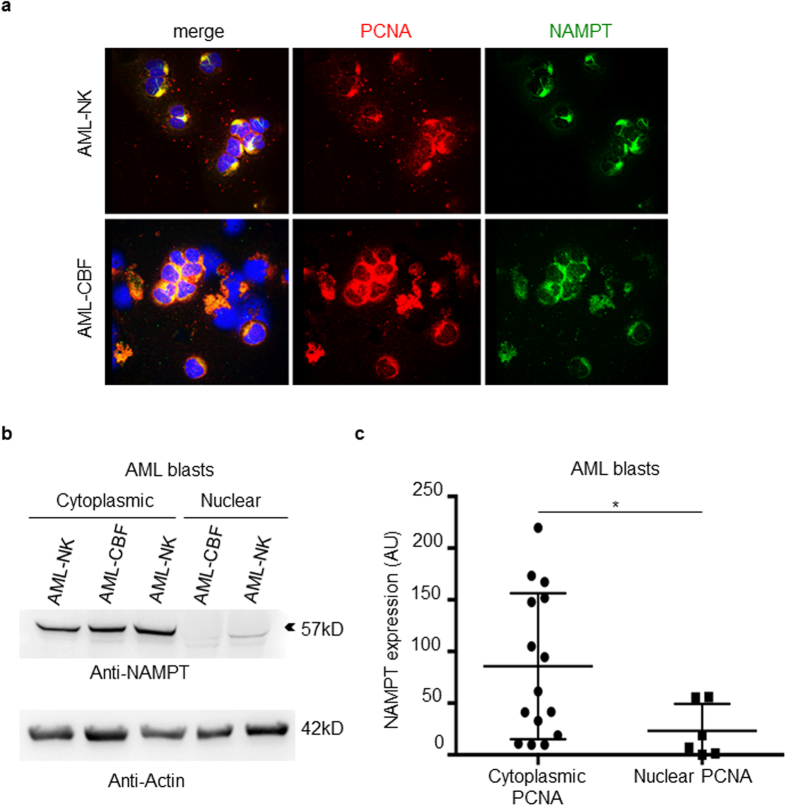
Analysis of NAMPT expression in blast from AML patients. (**a**) Colocalization between NAMPT (green) and PCNA (red) in blasts cells of AML patients after immunofluorescence labeling. This panel shows a representative experiment of three yielding the same results. (**b**) Western blot analysis of NAMPT expression in the cytosol of blasts from AML patient including three patients with cytoplasmic PCNA localization and two patients. (**c**) Results were quantified by densitometry and normalized to β-actin loading control for 15 AML patients with cytoplasmic PCNA versus 6 AML patients with nuclear PCNA. The data are mean ± SEM, *p < 0.05 (Mann-Whitney test).

Following LMB treatment, PCNA was relocated in the nuclei of cells from 3 AML patients with cytosolic PCNA localization (Fig. 6c), indicating that this cytosolic localization of PCNA in blast cells resulted from active nuclear export. Furthermore, p21-peptide-YIRS treatment of leukemic cells displaying cytoplasmic PCNA from 5 AML patients triggered apoptosis (Fig. 6d). Overall, these results indicated that a majority of AML patients at diagnosis display dysregulated PCNA export resulting in increased cytosolic PCNA levels, which may in turn favor AML cell survival. Confocal microscopy analysis of both NAMPT and PCNA revealed that these two proteins colocalized within the cytosol of primary leukemic cells (Fig. 7a). As observed in HL-60R cells, Western blot analysis provided evidence of a significant increased NAMPT expression in blasts from AML patients with a cytosolic PCNA localization compared to AML patients with a nuclear PCNA localization (Fig. 7b,c). Of note in this regard, the 57 kDa isoform of NAMPT expressed in HL-60R cells (Fig. 4b), was also prominent in blast cells from AML patients.

## Discussion

The notion that we previously described in mature neutrophils, that cytosolic PCNA promotes cell survival has been confirmed in HL-60 cells resistant to chemotherapy. From our present findings, we propose a new paradigm in which an increase in PCNA nuclear export results in a prominent cytosolic PCNA localization, which in turn builds a protein scaffold that dictates cell survival by enhancing their glycolytic metabolism and conferring chemotherapy resistance.

Our data provide unambiguous evidence that HL60-R cells harbor a prominently cytosolic PCNA compared with their sensitive counterparts and that the nuclear-to-cytosolic relocalization of PCNA in HL-60R cells involves CRM1-dependent nuclear export. Accordingly, inhibiting the PCNA nuclear export by LMB triggers caspase-dependent apoptosis in HL-60R but not in HL-60S cells. In multiple myeloma in which plasma cells display high levels of cytosolic PCNA, it was reported that a caspase-dependent apoptotic response could be induced by directly targeting PCNA with competing peptides^24^. Moreover, in human neuroblastoma cells, the association between procaspase-9 and cytosolic PCNA could be disrupted after S-nitrosylation of PCNA, in turn triggering apoptosis^25^. In our current study, we hypothesized that the retention of PCNA within the nucleus by LMB facilitated caspase activation and in turn potentiated apoptosis in HL-60R cells only. Our present data are consistent with the notion that CRM1 exerted antiapoptotic functions and was highly prognostic in AML^26^.

A plethora of interacting partners have been identified for nuclear PCNA. However little is known about its cytosolic interacting proteins reported by Naryzhny and Lee^27^. In the latter study, the authors used an overlay technique using recombinant PCNA to identified proteins containing a PCNA motif. This method does not allow to find PCNA-binding that would require a post-translational modification to bind to PCNA. Another major difference is that the assay was performed on a whole lysate in contrast to the cytosolic fraction that we have used in the current study. However, we have confirmed the interaction between PCNA and alpha-enolase (ENOA1) and with glyceraldehyde 3-phosphate (GAPDH) that were previously reported by Naryzhny and Lee. In contrast, the authors failed to detect an interaction between lactate deshydrogenase (LDH) and with pyruvate kinase (PK). Interestingly, using our co-immunoprecipitation protocol, we could find an interaction between endogenous PCNA and LDH and PKM2 only in HL-60S but not in HL-60R. This suggests that these interactions might be affected by some PCNA post-translational modifications which might also be influenced by the resistance to chemotherapy. Our proteomic analysis of the cytoplasmic PCNA interactome of HL-60S cells showed that several enzymes from the glycolysis pathway are associated with cytosolic PCNA. Notably, these interactions were different in HL-60S and in HL-60R cells, strongly suggesting that PCNA scaffold is adapted to the cellular metabolism to promote survival associated with chemotherapy resistance. We provide evidence for the first time for a direct interaction between NAMPT and PCNA, which resulted in a significant increase in the concentration of NAD+ in HL-60R cells that in turn acts to potentiate the glycolytic pathway, as previously demonstrated in other cell types^20^. Decreased intracellular NAD+ concentration and decreased glycolysis rate were observed in HL-60R cells treated with the p21-peptide-YIRS, which serves to disrupt cytoplasmic PCNA functions. However, our results show that the p21-peptide-YIRS did not suppress the direct interaction between NAMPT and PCNA measured by surface plasmon resonance. One possible interpretation is that NAMPT has two (or more) binding sites on PCNA and that the p21-peptide-YIRS cannot antagonize all the PCNA binding sites for NAMPT. In this context, when p21-peptide-YIRS and NAMPT are injected on PCNA immobilized on the support, both p21-peptide-YIRS and NAMPT can bind and the signal obtained is the sum of both. However, the p21 peptide could still disturb the function of NAMPT and affect NAD level. Altogether, our data provides evidence of a link between cytoplasmic PCNA and NAD-dependent metabolism. Interestingly, it has been reported that FK866, a small molecule inhibitor of NAMPT, can decrease leukemia cell viability but its relationship with cytosolic PCNA has yet to be investigated^28^,^29^. Notably, the increase NAD+ concentration could affect gene expression controlled by sirtuins, a NAD-dependent histone desacetylase^30^. It has recently been reported that high sirtuin 2 expression was associated with unfavorable prognostic^31^. We also provide evidence that both the glycolytic pathway and nuclear export are functionally linked in HL-60R cells as LMB significantly decreased lactate production. Conversely, glycolysis inhibition by 2-DG resulted in a disrupted PCNA export, presumably by reducing the cellular energy supply. It has been described previously that the subcellular compartimentalization of glycolytic enzymes^32^ and nuclear export^33^ can regulate the glycolysis flux. For instance, glucose availability regulates the subcellular distribution of karyopherins, which are proteins that control nucleocytoplasmic transport^34^. Collectively, our current results suggest that, in HL60-R cells, PCNA nuclear export is facilitated by an increased expression of CRM-1 and an increased glycolysis, which in turn promotes PCNA export. This ultimately results in a positive feedback loop and an accelerated and dysregulated glycolysis in HL-60R cells controlled by the PCNA scaffold.

AML represent a heterogeneous group of hematological disorders best categorized by their cytogenetic characteristics and mutational status^35^,^36^,^37^. Previous studies have shown that higher expression of PCNA in blast cells was correlated with blast cell percentage in myelodysplastic syndromes and in relapsed AML but no information on PCNA localization was provided^38^. We have studied a large set of primary AML samples and identified a predominantly cytosolic PCNA localization in 66.7% of these cases. This cytosolic PCNA localization along the normal myeloid pathway of differentiation is found only in mature neutrophils and never in normal CD34+ hematopoietic progenitors at the promyelocytic stage. In AML, cytoplasmic PCNA localization was observed at diagnosis, prior to chemotherapy induction, and did not correlate with cytogenetic characteristics. No difference was identified in the percentage of blast cells in the blood or bone marrow when comparing AML samples with cytoplasmic PCNA or with a nuclear/mixed pattern, suggesting that the proliferative status does not influence PCNA localization. While the exact reason for cytoplasmic PCNA localization in some patients remains unknown, it may result of hyperactive CRM1-dependent nuclear export. CRM1 inhibition triggered a caspase-dependent apoptosis pathway mainly in HL-60R cells, which is in keeping with the notion that PCNA partners are different between HL-60S and HL60-R cells.

Metabolic targeting of AML represents a new area of research^39^. Another illustration is the discovery of the increased production of 2-hydroxyglutarate (2-HG) and the recent development of specific inhibitors of isocitrate deshydrogenase gene mutants (IDH1/IDH2)^40^. More widely, inhibiting glutamine uptake by AML cells or glutaminolysis at different steps, represents a new challenge requiring further investigation to understand the molecular mechanisms that disturb these metabolic pathways in leukemic cells^41^,^42^. One salient feature of our data is the association between cytosolic PCNA and proteins from the glycolysis pathway that has been already identified as a critical pathway for cancer therapy^43^. Notably a recent metabolomics study has shown that a higher glycolytic activity in AML blast cells is associated with a poor prognosis^44^. The colocalization of NAMPT and PCNA within the cytoplasm, as well as increased expression of NAMPT has also been observed in primary AML samples with predominant cytoplasmic PCNA localization.

Our present data are highly suggestive that cytosolic PCNA is a key factor in shaping the energy metabolism that mediates the Warburg effect in AML. Most importantly, our results have uncovered a novel regulatory loop in the pro-survival activity of the cytosolic PCNA scaffold that links CRM1-dependent nuclear export and glycolysis in AML.

Targeting nuclear PCNA to inhibit cell proliferation in cancer by using competing peptides especially the p21 peptide has been well described^4^,^18^. It should be noted that in some cell types such as HeLa cell some PCNA-independent effects have been observed due to the presence of the RLIFS CDK-binding domain at the carboxy terminus of the p21 peptide^45^. In contrast, in p53 deficient cells (such as HL-60 cells) the p21 peptide mutated in its PCNA binding domain was inable to interfere with cell proliferation^46^.

The urgent need for innovative strategies to selectively eliminate AML cells should prompt us to carefully decipher the components of the cytosolic scaffold of PCNA^4^. For instance, targeting cytosolic PCNA in multiple myeloma cells using an APIM peptide has increased sensitivity to chemotherapy and induced apoptosis^24^. This should open novel avenues of investigation to specifically target key survival partners in leukemic cells and in other types of cancer cells since this scaffold appears to be a master element governing cell fates. Our finding that cytosolic PCNA could play a role in the increased survival of leukemic cells should offer new therapeutic possibilities if we can decipher the molecular and the biochemical features of cytosolic versus nuclear PCNA.

## Methods

### Blast cells from AML patients and myeloid cell lines

Bone marrow or peripheral blood samples with more than a 70% blast cell content were obtained from 69 patients with newly diagnosed AML: 19 were followed by Didier Bouscary from the Department of hematology of Cochin Hospital; their characteristics are listed in Supplementary Table 3; 26 were intermediate risk AML patients from the GOELAMS AML2006IR trial (NCT00860639) and 24 were CBF (core binding factor)-AML patients from the ALFA-GOELAMS AML 2006 trial (ref NCT00428558) whose characteristics are listed in Supplementary Tables 4 and 5. Handling, conditioning and storing of patients samples were performed by GOELAMStheque (N° BB-0033-00073), the tumor bank of the GOELAMS group, Cochin Hospital, Paris. Cells from AML patients were collected after obtaining written informed consent in accordance with the approval from the Cochin hospital and INSERM institutional ethics committees of the GOELAMS protocols whose numbers are mentioned above. All experimental methods were carried out in accordance with the Declaration of Helsinki. The GOELAMStheque benefits from an authorization of the Commission Nationale de l’Informatique et des Libertés (CNIL) reference #1559652.

All cell lines were cultured in RPMI Medium 1640 supplemented with 10% fetal bovine serum (FBS), L-glutamine (2 mM) and antibiotics (penicillin 100 U/ml and streptomycin at 100 *μ*g/ml). The daunorubicin-sensitive HL-60 (HL-60S) and daunorubicin-resistant HL60 (HL-60R) cell lines were kindly gifted by Dr Ruoping Tang (Hôtel Dieu Hospital, Paris, France)^47^. The K562 (kind gift of Pr Mahon, Bordeaux, France^48^) cells were obtained by *in vitro* passaging in progressively increasing doses of the drug to reach the final concentration of 1 μM doxorubicin (Sigma) and 1 μM imatinib (Sigma). When indicated, HL-60 cells were incubated with chemotherapy agents such as daunorubicin (1 μM), etoposide (10 μM) or cytarabine (10 μM), which were kindly provided by Dr. Marie Laure Brandely-Piat (Hôtel Dieu Hospital, Paris, France).

### Evaluation of cell viability and apoptosis

HL-60 cells were incubated with gliotoxin (1 μg/ml, Sigma) to trigger apoptosis via the mitochondrial pathway as gliotoxin associates with Bax^49^ and inhibits NF-kappa B^50^. Where indicated, HL-60 cells or blasts from AML patients were treated with the p21-peptide-YIRS, which specifically binds to PCNA interconnecting loop domain to disrupt PCNA interactions with partners^18^,^51^,^52^. The peptide (KRRQTSMTDFYHSKRRLIFSRYIRS, Genecust) corresponds to the p21/waf1 carboxy-terminus sequence (residues 141–160) and contains a RYIRS carboxy-terminal extension facilitating cell entry as previously described^53^. This peptide was used as a tool to disrupt the PCNA scaffold. A modified peptide (Mutated-P21) containing mutations to abrogate PCNA binding (KRRQTGETDFDHAKAALIFSRYIRS, Genecust) was used as a negative control, as previously described^9^. Where indicated, HL-60 or blast cells from AML patients were incubated with leptomycin B (LMB)^54^ at the indicated concentrations to inhibit CRM1-dependent nuclear export with or without the pan-caspase inhibitor Z-VAD(OMe)-FMK (50 μM, Bachem) or the RIP1 inhibitor necrostatin-1 (50 μM, Alexis Biochemicals). Cell viability was evaluated by either direct cell counts or flow cytometry after exclusion of trypan blue or 7-AAD-positive cells, respectively. Flow cytometry analysis of apoptosis was carried out by measuring phosphatidylserine externalization after Annexin-V (Miltenyi Biotec) and 7-AAD (BD biosciences) labeling, mitochondrial depolarization after DIOC6 labeling (Sigma) or percentage of cells in the subG1 phase after propidium iodide (Sigma) labeling as previously described^9^.

### Indirect immunofluorescence labeling and spinning disk

Immunofluorescence analysis in HL-60 and blast cells from leukemic patients was performed, as previously described^9^ using primary antibodies against PCNA (rabbit, Ab5, Calbiochem) or NAMPT/Visfatin (monoclonal mouse, Enzo Life Sciences). Nuclei were stained by Hoechst (Sigma, 2 μg/ml). P21 peptide analysis in HL-60 was performed after incubation of FITC-coupled p21-peptide-YIRS as previously described^9^. Slides were mounted using fluoprep medium and analyzed by confocal microscopy with a Leica TCS SP5 AOBS imaging microscope, model X63, with LAS AF version 1.8 software. Image stacks were acquired with 0.3 nm z step (63 × 1.4NA oil objective; Leica) using Yokogawa CSU X1 Spinning Disk (Yokogawa, Tokyo, Japan) coupled with a DMI6000B Leica microscope (Leica Microsystems Gmbh, Wetzlar, Germany). Acquisitions were made with MetaMorph 7 software. Image analysis and quantification of the intensity of nuclear PCNA fluorescence was performed using ImageJ (http://imagej.nih.gov/ij/). To establish the intensity of nuclear PCNA fluorescence, an automatic threshold was performed and a Hoechst binary mask was used to quantify the labelled area within the nucleus.

### Subcellular fractionation and western blot analysis

PCNA expression was evaluated by western blot analysis in HL-60 cells lyzed in PBS supplemented with 1% Triton-X100 and anti-proteinases or in the nuclear fraction after cell potterization, as previously described^55^. Nitrogen cavitation was used to obtain protease-free cytosolic fractions, as previously described^56^. NAMPT expression was evaluated by western blot analysis in blast cells after sonication to obtain the cytosolic fraction^9^. Western blot analysis of PCNA (mouse monoclonal PC10), lamin B1 (rabbit polyclonal Santa Cruz), CRM-1 (rabbit polyclonal Santa Cruz), NAMPT/Visfatin (mouse monoclonal Enzo life sciences), hexokinase 1 (rabbit polyclonal Abcam), caspase-8 (mouse monoclonal Alexis), cleaved caspase-3 and caspase-9 (rabbit Cell signaling), caspase 3 (mouse monoclonal Santa Cruz) were carried out as previously described^9^ and β-actin (rabbit polyclonal Sigma) expression was used as a loading control.

### SiRNA transfection

To knock down PCNA expression, HL-60R cells were transfected with 1 *μ*M of small-interfering RNA (siRNA, Ambion) as previously described^9^.

### Characterization of molecular interaction by Surface Plasmon Resonance (SPR)

Surface plasmon resonance measurements were performed using a Biacore T200 system (GE Healthcare) designed to calculate affinity and kinetic parameters. A sensor chip covered with a dextran layer (CM5) was used in this system to calculate these parameters. PCNA was covalently immobilized on the CM5 sensor chip using 10 mM Hepes, 150 mM NaCl pH 7.4 containing 5 mM CaCl2 and 0.05% P20 as the running buffer. NAMPT (25–400 nM) was injected at 20 μl/min for 120 s over immobilized PCNA and over a control flow cell subjected to the coupling steps without PCNA (activated–deactivated surface). The running buffer for the interaction was 50 mM Tris, 150 mM NaCl pH 7.4 containing 2 mM CaCl2. Kinetic titration, referred to as single-cycle kinetics, was used to collect binding data for kinetic analysis. This method involved sequential injections of several analyte concentrations over the ligand immobilized on the sensor chip surface without a regeneration step between successive injections. The association (ka) and dissociation rate (kd) constants and the equilibrium dissociation constant (KD) were calculated using the T200 Biaevaluation software. For analysis of the p21 peptide effect, p21 (2 μM), NAMPT (120 nM) or a mixture of both at these same concentrations were injected at 20 μl/min for 100 s over immobilized PCNA and dissociation was analyzed for 400 s. The specific binding signal was obtained by subtracting the background signal, routinely obtained by injection of the sample over an activated–deactivated surface as previously described^23^.

### Evaluation of metabolism in HL-60 cells

Total intracellular NAD was measured using the Biovision assay kit according to the manufacturer’s instructions. Briefly, 1.5 ml containing 3.75 × 10^5^ of cells were incubated at 37 °C in 5% CO_2_ with the p21-peptide-YIRS (10 μM, 25 μM) or 3-bromopyruvate (3-BrP) (20 μg/ml, Calbiochem) for 6 hours. Metabolism was evaluated by measuring the extracellular acidification rate (ECAR) and the oxygen consumption rate (OCR) reflecting the glycolysis and the mitochondrial oxidative phosphorylation pathway, respectively with an XF96 Extracellular Flux analyzer (Seahorse Bioscience). Cells were incubated with or without p21-peptide-YIRS (5 μM, 10 μM), mutated p21-peptide (Mut-P21, 10 μM) or LMB (10 ng/ml, 20 ng/ml). The cells were then plated into XF96 polystyrene cell culture dishes coated with 3.5 μg/cm^2^ Corning Cell-Tak (Dutscher). Briefly, 0.2 × 10^5^ HL-60 cells/well were resuspended in 140 μl of XF assay medium containing 2 mM L-glutamine and placed in XF96 culture plates. While sensor cartridges were calibrated, cell plates were incubated in a 37 °C/non CO_2_ incubator for 60 min prior to the start of the assay. All experiments were performed at 37 °C. ECAR and OCR response were measured at baseline and after adding D-glucose at two different concentrations (G1 at 10 mM and G2 at 20 mM, Sigma), oligomycin (1 μg/ml, Clinisciences) and 2-deoxy-D-glucose (50 mM, Clinisciences). Results were also expressed using the area under the curve.

L-lactate was measured in the culture medium with colorimetric L-Lactate Assay Kit (Abcam, #ab65331) according to the manufacturer’s instructions. Briefly, 0.25 × 10^6^ cells were resuspended in 3 ml of RPMI containing 0.5% of FBS for 6 hours and the medium was collected. Measurement of intracellular glucose-6-phosphate (G6P) was performed using the G6P Assay Kit (Abcam, #ab83426) as described by the manufacturer. Briefly, 10^7^ cells were cultured in 40 ml of RPMI in the presence of 0.5% of FBS for 24 hours. Cells were sonicated on ice for 10 s using a Soniprep 150 Plus Ultrasonic Disintegrator (MSE) at the lowest setting, centrifuged for 10 min at 20,000 g at 4 °C and the G6P level was determined in the supernatant.

### Identification of PCNA partners by mass spectrometry

See Supplementary Methods for further details^56^,^57^.

### Statistical analysis

Statistical analysis was performed using the Statview™ software package. Comparisons were made using the Student’s *t* test, multicomparison ANOVA or the Mann & Whitney test as indicated. Differences were considered significant at P < 0.05.

## Additional Information

**How to cite this article**: Ohayon, D. *et al*. Cytoplasmic proliferating cell nuclear antigen connects glycolysis and cell survival in acute myeloid leukemia. *Sci. Rep.* **6**, 35561; doi: 10.1038/srep35561 (2016).

## Supplementary Material

Supplementary Information

## References

[b1] Indiani C. & O’Donnell M. The replication clamp-loading machine at work in the three domains of life. Nat Rev Mol Cell Biol 7, 751–761 (2006). 16955075 10.1038/nrm2022

[b2] Moldovan G. L., Pfander B. & Jentsch S. PCNA, the maestro of the replication fork. Cell 129, 665–679 (2007). 17512402 10.1016/j.cell.2007.05.003

[b3] Maga G. & Hubscher U. Proliferating cell nuclear antigen (PCNA): a dancer with many partners. J Cell Sci 116, 3051–3060 (2003). 12829735 10.1242/jcs.00653

[b4] Wang S. C. PCNA: a silent housekeeper or a potential therapeutic target? Trends Pharmacol Sci 35, 178–186 (2014). 24655521 10.1016/j.tips.2014.02.004

[b5] Witko-Sarsat V., Pederzoli-Ribeil M., Hirsch E., Sozzani S. & Cassatella M. A. Regulating neutrophil apoptosis: new players enter the game. Trends Immunol 32, 117–124 (2011). 21317039 10.1016/j.it.2011.01.001

[b6] Geering B. & Simon H. U. Peculiarities of cell death mechanisms in neutrophils. Cell Death Differ 18, 1457–1469 (2011). 21637292 10.1038/cdd.2011.75 PMC3178425

[b7] Altznauer F. . Inflammation-associated cell cycle-independent block of apoptosis by survivin in terminally differentiated neutrophils. J Exp Med 199, 1343–1354 (2004). 15148334 10.1084/jem.20032033 PMC2211817

[b8] Rossi A. G. . Cyclin-dependent kinase inhibitors enhance the resolution of inflammation by promoting inflammatory cell apoptosis. Nat Med 12, 1056–1064 (2006). 16951685 10.1038/nm1468

[b9] Witko-Sarsat V. . Proliferating cell nuclear antigen acts as a cytoplasmic platform controlling human neutrophil survival. J Exp Med 207, 2631–2645 (2010). 20975039 10.1084/jem.20092241 PMC2989777

[b10] Martin C. . Neutrophil-expressed p21/waf1 Favors Inflammation Resolution in Pseudomonas aeruginosa Infection. Am J Respir Cell Mol Biol (2015). 10.1165/rcmb.2015-0047OC 26517580

[b11] Greenlee-Wacker M. C. . Phagocytosis of Staphylococcus aureus by human neutrophils prevents macrophage efferocytosis and induces programmed necrosis. J Immunol 192, 4709–4717 (2014). 24729616 10.4049/jimmunol.1302692 PMC4011196

[b12] De Chiara A. D., Pederzoli-Ribeil M., Burgel P. R., Danel C. & Witko-Sarsat V. Targeting cytosolic proliferating cell nuclear antigen in neutrophil-dominated inflammation. Front Immunol 3, 311 (2012). 23181059 10.3389/fimmu.2012.00311 PMC3501000

[b13] Witko-Sarsat V. & Ohayon D. Proliferating cell nuclear antigen in neutrophil fate. Immunol Rev 273, 344–356 (2016). 27558345 10.1111/imr.12449

[b14] Bouayad D. . Nuclear-to-cytoplasmic relocalization of the proliferating cell nuclear antigen (PCNA) during differentiation involves a chromosome region maintenance 1 (CRM1)-dependent export and is a prerequisite for PCNA antiapoptotic activity in mature neutrophils. J Biol Chem 287, 33812–33825 (2012). 22846997 10.1074/jbc.M112.367839 PMC3460476

[b15] De Chiara A. . Characterization of cytosolic proliferating cell nuclear antigen (PCNA) in neutrophils: antiapoptotic role of the monomer. J Leukocyte Biol 94, 723–731 (2013). 23825390 10.1189/jlb.1212637

[b16] Grimwade D., Ivey A. & Huntly B. J. Molecular landscape of acute myeloid leukemia in younger adults and its clinical relevance. Blood 127, 29–41 (2016). 26660431 10.1182/blood-2015-07-604496 PMC4705608

[b17] Testa U. & Riccioni R. Deregulation of apoptosis in acute myeloid leukemia. Haematologica 92, 81–94 (2007). 17229639 10.3324/haematol.10279

[b18] Warbrick E. The puzzle of PCNA’s many partners. Bioessays 22, 997–1006 (2000). 11056476 10.1002/1521-1878(200011)22:11<997::AID-BIES6>3.0.CO;2-#

[b19] Garten A., Petzold S., Korner A., Imai S. & Kiess W. Nampt: linking NAD biology, metabolism and cancer. Trends Endocrin Met 20, 130–138 (2009). 10.1016/j.tem.2008.10.004 PMC2738422 19109034

[b20] Tan B. . Pharmacological inhibition of nicotinamide phosphoribosyltransferase (NAMPT), an enzyme essential for NAD+ biosynthesis, in human cancer cells: metabolic basis and potential clinical implications. J Biol Chem 288, 3500–3511 (2013). 23239881 10.1074/jbc.M112.394510 PMC3561569

[b21] Wang T. . Structure of Nampt/PBEF/visfatin, a mammalian NAD+ biosynthetic enzyme. Nat Struct Mol Biol 13, 661–662 (2006). 16783373 10.1038/nsmb1114

[b22] Revollo J. R., Grimm A. A. & Imai S. The NAD biosynthesis pathway mediated by nicotinamide phosphoribosyltransferase regulates Sir2 activity in mammalian cells. J Biol Chem 279, 50754–50763 (2004). 15381699 10.1074/jbc.M408388200

[b23] Terrasse R. . Human and pneumococcal cell surface glyceraldehyde-3-phosphate dehydrogenase (GAPDH) proteins are both ligands of human C1q protein. J Biol Chem 287, 42620–42633 (2012). 23086952 10.1074/jbc.M112.423731 PMC3522263

[b24] Muller R. . Targeting proliferating cell nuclear antigen and its protein interactions induces apoptosis in multiple myeloma cells. PLoS One 8, e70430 (2013). 23936203 10.1371/journal.pone.0070430 PMC3729839

[b25] Yin L. . The S-nitrosylation status of PCNA localized in cytosol impacts the apoptotic pathway in a Parkinson’s disease paradigm. PLoS One 10, e0117546 (2015). 25675097 10.1371/journal.pone.0117546 PMC4326459

[b26] Kojima K. . Prognostic impact and targeting of CRM1 in acute myeloid leukemia. Blood 121, 4166–4174 (2013). 23564911 10.1182/blood-2012-08-447581 PMC3656451

[b27] Naryzhny S. N. & Lee H. Proliferating cell nuclear antigen in the cytoplasm interacts with components of glycolysis and cancer. FEBS letters 584, 4292–4298 (2010). 20849852 10.1016/j.febslet.2010.09.021

[b28] Zoppoli G. . Potent synergistic interaction between the Nampt inhibitor APO866 and the apoptosis activator TRAIL in human leukemia cells. Exp Hematol 38, 979–988 (2010). 20696207 10.1016/j.exphem.2010.07.013

[b29] Wosikowski K. . WK175, a novel antitumor agent, decreases the intracellular nicotinamide adenine dinucleotide concentration and induces the apoptotic cascade in human leukemia cells. Cancer Res 62, 1057–1062 (2002). 11861382

[b30] Saunders L. R. & Verdin E. Sirtuins: critical regulators at the crossroads between cancer and aging. Oncogene 26, 5489–5504 (2007). 17694089 10.1038/sj.onc.1210616

[b31] Deng A., Ning Q., Zhou L. & Liang Y. SIRT2 is an unfavorable prognostic biomarker in patients with acute myeloid leukemia. Sci Rep 6, 27694 (2016). 27291931 10.1038/srep27694 PMC4904374

[b32] Brown V. M. . A novel CRM1-mediated nuclear export signal governs nuclear accumulation of glyceraldehyde-3-phosphate dehydrogenase following genotoxic stress. J Biol Chem 279, 5984–5992 (2004). 14617633 10.1074/jbc.M307071200

[b33] Schwoebel E. D., Ho T. H. & Moore M. S. The mechanism of inhibition of Ran-dependent nuclear transport by cellular ATP depletion. J Cell Biol 157, 963–974 (2002). 12058015 10.1083/jcb.200111077 PMC2174045

[b34] Huang H. Y. & Hopper A. K. Separate responses of karyopherins to glucose and amino acid availability regulate nucleocytoplasmic transport. Mol Biol Cell 25, 2840–2852 (2014). 25057022 10.1091/mbc.E14-04-0948 PMC4161518

[b35] Dohner H. . Diagnosis and management of acute myeloid leukemia in adults: recommendations from an international expert panel, on behalf of the European LeukemiaNet. Blood 115, 453–474 (2010). 19880497 10.1182/blood-2009-07-235358

[b36] Hourigan C. S. & Karp J. E. Personalized therapy for acute myeloid leukemia. Cancer Discov 3, 1336–1338 (2013). 24327695 10.1158/2159-8290.CD-13-0832 PMC3880545

[b37] Forthun R. B., Hinrichs C., Dowling T. H., Bruserud O. & Selheim F. The Past, Present and Future Subclassification of Patients with Acute Myeloid Leukemia. Curr Pharm Biotechnol 17, 6–19 (2016). 26343130 10.2174/1389201016666150907113653

[b38] Staber P. B. . Common alterations in gene expression and increased proliferation in recurrent acute myeloid leukemia. Oncogene 23, 894–904 (2004). 14749762 10.1038/sj.onc.1207192

[b39] Abdel-Wahab O. & Levine R. L. Metabolism and the leukemic stem cell. J Exp Med 207, 677–680 (2010). 20368582 10.1084/jem.20100523 PMC2856035

[b40] Gross S. . Cancer-associated metabolite 2-hydroxyglutarate accumulates in acute myelogenous leukemia with isocitrate dehydrogenase 1 and 2 mutations. J Exp Med 207, 339–344 (2010). 20142433 10.1084/jem.20092506 PMC2822606

[b41] Willems L. . Inhibiting glutamine uptake represents an attractive new strategy for treating acute myeloid leukemia. Blood 122, 3521–3532 (2013). 24014241 10.1182/blood-2013-03-493163 PMC3829119

[b42] Jacque N. . Targeting glutaminolysis has antileukemic activity in acute myeloid leukemia and synergizes with BCL-2 inhibition. Blood 126, 1346–1356 (2015). 26186940 10.1182/blood-2015-01-621870 PMC4608389

[b43] Hamanaka R. B. & Chandel N. S. Targeting glucose metabolism for cancer therapy. J Exp Med 209, 211–215 (2012). 22330683 10.1084/jem.20120162 PMC3280882

[b44] Chen W. L. . A distinct glucose metabolism signature of acute myeloid leukemia with prognostic value. Blood 124, 1645–1654 (2014). 25006128 10.1182/blood-2014-02-554204 PMC5726328

[b45] Mattock H., Lane D. P. & Warbrick E. Inhibition of cell proliferation by the PCNA-binding region of p21 expressed as a GFP miniprotein. Exp Cell Res 265, 234–241 (2001). 11302688 10.1006/excr.2001.5160

[b46] Cayrol C., Knibiehler M. & Ducommun B. p21 binding to PCNA causes G1 and G2 cell cycle arrest in p53-deficient cells. Oncogene 16, 311–320 (1998). 9467956 10.1038/sj.onc.1201543

[b47] Tang R. . P-gp activity is a critical resistance factor against AVE9633 and DM4 cytotoxicity in leukaemia cell lines, but not a major mechanism of chemoresistance in cells from acute myeloid leukaemia patients. BMC Cancer 9, 199 (2009). 19549303 10.1186/1471-2407-9-199 PMC2708190

[b48] Mahon F. X. . Selection and characterization of BCR-ABL positive cell lines with differential sensitivity to the tyrosine kinase inhibitor STI571: diverse mechanisms of resistance. Blood 96, 1070–1079 (2000). 10910924

[b49] Pardo J. . The mitochondrial protein Bak is pivotal for gliotoxin-induced apoptosis and a critical host factor of Aspergillus fumigatus virulence in mice. J Cell Biol 174, 509–519 (2006). 16893972 10.1083/jcb.200604044 PMC2064257

[b50] Ward C. . NF-kappaB activation is a critical regulator of human granulocyte apoptosis *in vitro*. J Biol Chem 274, 4309–4318 (1999). 9933632 10.1074/jbc.274.7.4309

[b51] Warbrick E. A functional analysis of PCNA-binding peptides derived from protein sequence, interaction screening and rational design. Oncogene 25, 2850–2859 (2006). 16407840 10.1038/sj.onc.1209320 PMC2699888

[b52] Kroker A. J. & Bruning J. B. p21 Exploits Residue Tyr151 as a Tether for High-Affinity PCNA Binding. Biochemistry 54, 3483–3493 (2015). 25972089 10.1021/acs.biochem.5b00241

[b53] Dong C., Li Q., Lyu S. C., Krensky A. M. & Clayberger C. A novel apoptosis pathway activated by the carboxyl terminus of p21. Blood 105, 1187–1194 (2005). 15466931 10.1182/blood-2004-06-2188

[b54] Nishi K. . Leptomycin B targets a regulatory cascade of crm1, a fission yeast nuclear protein, involved in control of higher order chromosome structure and gene expression. J Biol Chem 269, 6320–6324 (1994). 8119981

[b55] Pederzoli M., Kantari C., Gausson V., Moriceau S. & Witko-Sarsat V. Proteinase-3 induces procaspase-3 activation in the absence of apoptosis: potential role of this compartmentalized activation of membrane-associated procaspase-3 in neutrophils. J Immunol 174, 6381–6390 (2005). 15879139 10.4049/jimmunol.174.10.6381

[b56] Borregaard N., Heiple J. M., Simons E. R. & Clark R. A. Subcellular localization of the b-cytochrome component of the human neutrophil microbicidal oxidase: translocation during activation. J Cell Biol 97, 52–61 (1983). 6408102 10.1083/jcb.97.1.52 PMC2112494

[b57] Poullet P., Carpentier S. & Barillot E. myProMS, a web server for management and validation of mass spectrometry-based proteomic data. Proteomics 7, 2553–2556 (2007). 17610305 10.1002/pmic.200600784

