# Supplementary material for: Cytoplasmic proliferating cell nuclear antigen connects glycolysis and cell survival in acute myeloid leukemia
Source: Sci Rep. 2016 Oct 19;6:35561. doi: 10.1038/srep35561 (PMC5069676; doi:10.1038/srep35561)
Supplement: Supplementary Information [file srep35561-s1.pdf]

Cytoplasmic proliferating cell nuclear antigen connects glycolysis and cell survival in acute myeloid leukemia

Delphine Ohayon[1,2,3,4], Alessia De Chiara[1,2,3], Nicolas Chapuis[1,2,3,5,6], Céline Candalh[1,2,3], Julie Mocek[1,2,3], Jean-Antoine Ribeil[7], Lamya Haddaoui[1,2,3,6], Norbert Ifrah[6,8], Olivier Hermine[9], Frédéric Bouillaud[1,2,3], Philippe Frachet[10,11], Didier Bouscary[1,2,3,5,6] and Véronique Witko-Sarsat[1,2,3,4,*]

1 INSERM U1016, Paris, France

2 Institut Cochin, Université Paris Descartes, Faculté de Médecine Sorbonne Paris Cité, Paris, France.

3 CNRS UMR 8104, Paris, France

4 Center of Excellence, Labex Inflamex, 75014 France

5 Hematology Department, Cochin Hospital, AP-HP, Paris, France.

6 FILO : French Innovative Leukemia Organization (GOELAMS), CHU Bretonneau, TOURS France

7 Biotherapy Department, Necker Hospital, Paris, France.

8 Hematology Department CHU & UMR INSERM U892/CNRS6299, Université d'Angers, France

9 Hematology Department, CNRS UMR 8143, Université Paris Descartes, Necker Hospital, France

10 Institut de Biologie Structurale, Centre Etude Atomique, Grenoble, France

11 Université Grenoble Alpes, CNRS, UMR 5075, Grenoble, France;

Supplementary Methods:

Identification of PCNA partners by mass spectrometry

Rabbit polyclonal anti-PCNA Ab5 antibody (20 μg) or control IgG were crosslinked to 50 μl of protein G sepharose beads (Pierce) using 100 μl DMP (20 mM dimethyl pimelimidate, Pierce) in the cross-linking buffer (carbonate buffer 200 mM, pH 9) for 30 min according to the manufacturer's instructions. Cross linking reaction was stopped by a 15 min incubation in 100 μl of Tris 50 mM, followed by one lavage with 500 μl of glycine solution (50 mM pH 2,7). Proteins (200 μg) from cytosols from HL-60S and HL-60R cells obtained by nitrogen cavitation as previously described [56] were added to sepharose beads crosslinked with anti-PCNA antibody and incubated for 2 h at 4°C. At the end of this incubation, proteins bound to the beads were recovered after extensive washing as previously described [9]. Proteins obtained by immunoprecipitation were denatured at 95°C in reducing conditions with Laemmli buffer. Supernatants were loaded onto an SDS-PAGE gel for a short migration and proteins were trypsin-digested in-gel. For LC-MS protein analysis, peptides were concentrated, washed and analyzed using a reverse phase C18 column on a u3000 nanoHPLC hyphenated to a Linear Trap Quadrupole-Orbitrap mass spectrometer (Thermo). LTQ MS/MS CID spectra were acquired from up to the 20 most abundant ions detected in the Orbitrap MS scan. Protein identifications were performed with the Proteome discoverer 1.3 (Thermo) and with Mascot (www.matrixscience.com). Separate analysis was conducted and compared using MyPROMS software [57].

Supplementary Figures:

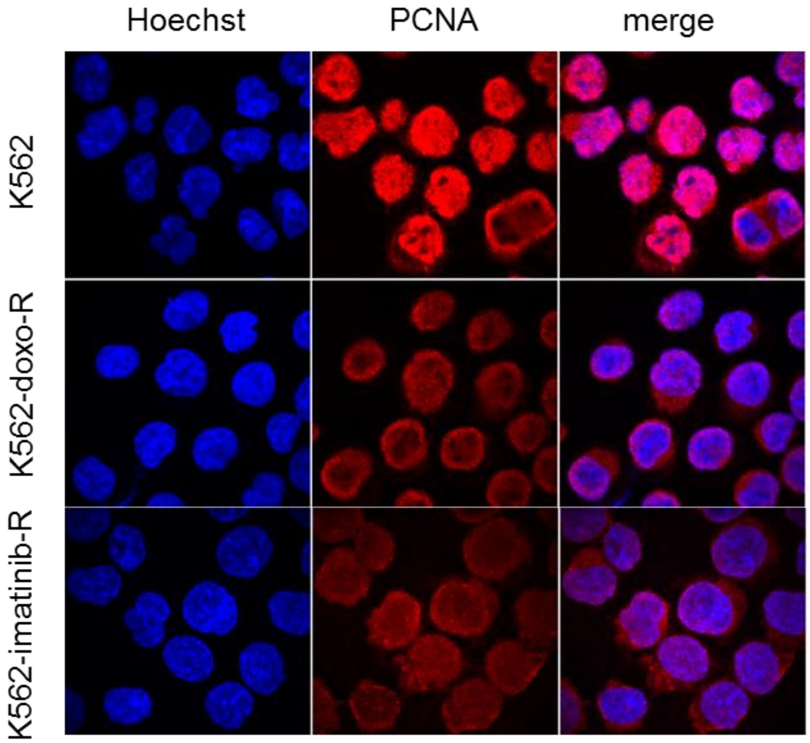

Supplementary Figure S1: Cytoplasmic localization of PCNA in doxorubicin and imatinib resistant (K562) cells.

Immunofluorescence analysis of PCNA localization in K562 treated or not with doxorubicin or imatinib. Immunolabeling was performed using Ab5 rabbit polyclonal antibody and nuclei visualized by Hoechst (2µg/ml). Cells were analyzed by confocal microscopy and this representative experiment was performed 3 times with identical results.

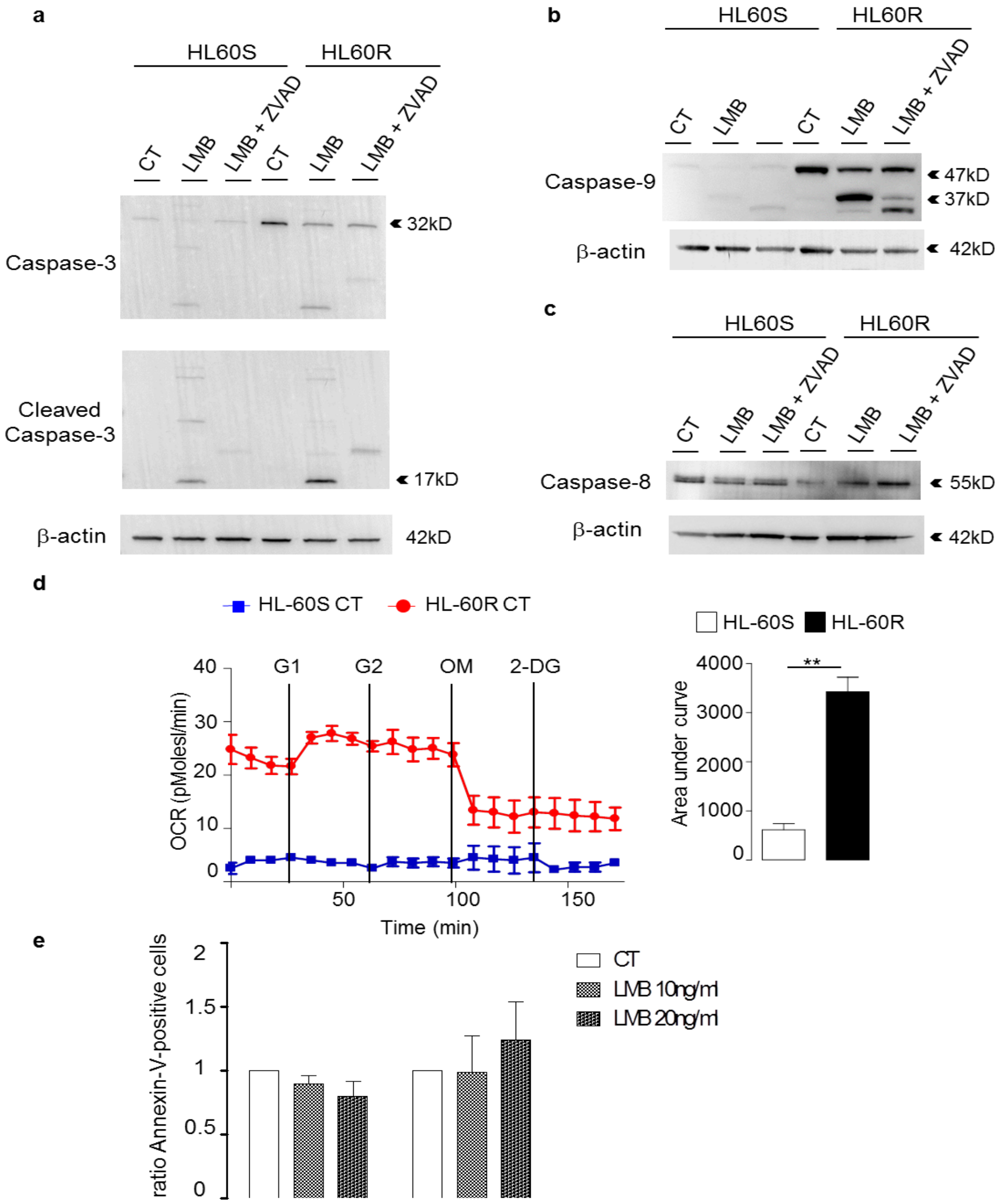

Supplementary Figure S2: Functional characterization of HL-60 cells.
(a-b-c) Western blot analysis of caspase-3 (a), caspase-9 (b) and caspase-8 (c) in HL-60 cells cultured for 16 h at 37°C in the absence or in the presence of LMB at 10 ng/ml and Z-VAD(OMe)-FMK at (50 µM). Cells ($5x10^6$) were lyzed in 1% Triton-X100 buffer and caspase-3, caspase-9 and caspase-8 proteins analyzed by Western blot (50 µg/lane). (d) Kinetic oxygen consumption rate (OCR) response of HL-60 cells at baseline and after adding D-Glucose at two different concentrations (G1 at 10 mM and G2 at 20 mM), oligomycin (OM at 1 µg/ml) and 2-Deoxy-D-glucose (2-DG at 50 mM). The data are mean ± SEM of three independent experiments performed in quintuplets, **$p < 0.01$ (Student's *t* test) (e) Effect of LMB on the apoptosis of HL-60S and HL-60R. HL-60 cells cultured for 6h at 37°C in the absence or presence of LMB (10 or 20 ng/ml). The percentages of apoptotic cells were measured by annexin-V^+ using flow cytometry and compared to control.

Supplementary Tables:

Supplementary Table 1: Identification of proteins by mass spectrometry in the material eluted from the anti-PCNA co-immunoprecipitation.

Swiss Prot	Protein Name	Gene name	ID	Peptide ratio
SYDC_HUMAN	Aspartate-tRNA ligase, cytoplasmic	DARS	P14868	10
DX39A_HUMAN	ATP-dependent RNA helicase DDX39A	DDX39A	O00148	10
ENOA_HUMAN	Alpha-enolase	ENO1	P06733	-3,003
SYEP_HUMAN	Bifunctional glutamate/proline--tRNA ligase	EPRS	P07814	10
**G3P_HUMAN**	**Glyceraldehyde-3-phosphate dehydrogenase**	**GAPDH**	**P04406**	**1.67**
GELS_HUMAN	Gelsolin	GSN	P06396	1
HS90A_HUMAN	Heat shock protein HSP 90-alpha	HSP90AA1	P07900	1.5
HS90B_HUMAN	Heat shock protein HSP 90-beta	HSP90AB1	P08238	-1.064
HSP7C_HUMAN	Heat shock cognate 71 kDa protein	HSPA8	P11142	-1.802
CH60_HUMAN	60 kDa heat shock protein, mitochondrial	HSPD1	P10809	4
IPO5_HUMAN	Importin-5	IPO5	O00410	10
IMB1_HUMAN	Importin subunit beta-1	KPNB1	Q14974	1
**LDHA_HUMAN**	**L-lactate dehydrogenase A chain**	**LDHA**	**P00338**	**3.5**
**LDHB_HUMAN**	**L-lactate dehydrogenase B chain**	**LDHB**	**P07195**	**10**
**NAMPT_HUMAN**	**Nicotinamide phosphoribosyltransferase**	**NAMPT**	**P43490**	**-10**
PCNA_HUMAN	Proliferating cell nuclear antigen	PCNA	P12004	-1.064
PDC6I_HUMAN	Programmed cell death 6-interacting protein	PDCD6IP	Q8WUM4	10
**KPYR_HUMAN**	**Pyruvate kinase PKLR**	**PKLR**	**P30613**	**10**
**KPYM_HUMAN**	**Pyruvate kinase PKM**	**PKM**	**P14618**	**10**
**PMM2_HUMAN**	**Phosphomannomutase 2**	**PMM2**	**O15305**	**2.5**
PON1_HUMAN	Serum paraoxonase/arylesterase 1	PON1	P27169	-3.003
TRFE_HUMAN	Serotransferrin	TF	P02787	-2
TKT_HUMAN	Transketolase	TKT	P29401	10
UBP14_HUMAN	Ubiquitin carboxyl-terminal hydrolase 14	USP14	P54578	10
SYYC_HUMAN	Tyrosine-tRNA ligase, cytoplasmic	YARS	P54577	10
1433Z_HUMAN	14-3-3 protein zeta/delta	YWHAZ	P63104	-2

Immunoprecipitation of PCNA was performed in cytosols from HL-60S and HL-60R cells and proteins associated with PCNA were identified by mass spectrometry. The peptide numbers were determined for either HL-60S or HL-60R and the peptide ratio was calculated. A negative ratio was arbitrary given when the number of peptides was greater in the HL-60R cells than in the HL-60S. When a protein was not detected in either HL-60S or HL-60R an arbitrary ratio of -10 or 10, respectively, was given. This representative experiment has been performed two times with similar results. The enzymes related to the glycolysis pathway are indicated in bold.

Supplementary Table 2: Kinetics and affinity of PCNA-NAMPT interaction.

Immobilized ligand/Soluble analyte	**PCNA/NAMPT**					
2-state model	k_{a1} (M^{-1} s^{-1})	k_{d1} (s^{-1})	k_{a2} (s^{-1})	k_{d2} (s^{-1})	**K_D (M)**	***Chi^2***
	8104	1.5×10^{-2}	7.0×10^{-3}	7.5×10^{-4}	**1.8×10^{-7}**	**0.148**

Binding of NAMPT (25-400 nM) was measured as described under *Methods*.
Values were calculated for 2-state reaction (Biaevaluation software). In this model, the association (k_{a1}, $k_{a2)}$ and dissociation (k_{d1}, k_{d2}) rate constants were determined by global fitting. The dissociation constant K_D was determined from the (k_{d1} / k_{a1}) * (k_{d2} / k_{a2}) ratios.

Supplementary Table 3: Clinical characteristics of the patients from the Departmental of Hematology of Cochin Hospital (n= 19).

BM: bone marrow, CR: complete remission, MDS: myelodysplastic syndromes, ET: Essential Thrombocythaemia, NA : not available.

Patient	AML type	BM or blood sample	% of blast cells	Caryotype	PCNA localization
1	AML4	Blood	77	46,XY,del(7)(q21q35),inv(16)(p13q22)[20]	cytoplasmic
2	AML2	Blood	60	NA	nuclear
3	AML4	BM	56	46,XY,inv(16)(p13q22)[18]/46,XY[2]	cytoplasmic
4	AML2	BM	76	46,XY[26]	cytoplasmic
5	AML2	BM	50	46,XY,inv(16)(p13q22)[15]/46,idem,del(13)(q13q21)[4]/47,idem,+22[1]	nuclear
6	AML1	BM	98	46,XY[20]	nuclear
7	AML post ET	Blood	98	46,XX[20]	nuclear
8	AML1	BM	75	46,XY,del(9)(q21q32)[10]/46,XY[10]	cytoplasmic
9	AML1	BM	75	46,XY[20]	cytoplasmic
10	AML5	BM	95	NA	cytoplasmic
11	AML4	BM	45	46,XX[20]	cytoplasmic
12	AML4	BM	43	NA	cytoplasmic
13	AML post MDS	BM	59	46,XY[20]	nuclear
14	AML post MDS	Blood	75	46,XX,del(5)(q14 15q34) [20]	cytoplasmic
15	AML1	BM	93	46,XX[20]	cytoplasmic
16	AML4	Blood	79	NA	nuclear
17	AML4	BM	81	46,XY[20]	cytoplasmic
18	AML5	BM	87	46,XY[20]	nuclear
19	AML1	BM	95	46,XY[20]	nuclear

Supplementary Table 4: Clinical characteristics of the patients from GOELAMStheque with normal karyotype (NK)-AML (n=26).

BM: bone marrow, CR: complete remission, MDS: myelodysplastic syndromes, ET: Essential Thrombocythaemia, NA : not available.

Patient	BM or blood sample	% of blast cells	CR achieved with one course of treatment	Early disease relapse (<12months)	Time between diagnosis and relapse (month)	Caryotype	PCNA localization
NK 1	BM	76	Yes	✞1m		46,XX [25]	nuclear
NK 2	BM	89	No	No		46,XX[25]	cytoplasmic
NK 3	BM	84	No	Yes	6	46,XY[20]	nuclear
NK 4	BM	81	✞<15d	✞<15d		46,XX[39]	cytoplasmic
NK 5	Blood	84	Yes	No		46,XY,del(14)(q21q31)[2]/45,idem-Y[18]	cytoplasmic
NK 6	BM	84	No	✞5m		46,XX[20]	cytoplasmic
NK 7	Blood	80	Yes	NA		46,XY[20]	cytoplasmic
NK 8	BM	82	Yes	Yes	7	46,XX[24]	cytoplasmic
NK 9	BM	76	No	No		46,XX[25]	nuclear
NK 10	Blood	80	Yes	No		46,XY[9]/46,XYt(8;17;21)(q23;p12;q22)[10]	cytoplasmic
NK 11	Blood	68	Yes	No		46,XX[53]	nuclear
NK 12	Blood	78	Yes	No		46, XY[26]	nuclear
NK 13	BM	99	Yes	✞5m		46,XX[20]	cytoplasmic
NK 14	BM	92	No	NA		46,XY[25]	cytoplasmic
NK 15	BM	85	Yes	✞10m		47,XY,+8,der(16)t(1;16)(q11;q11)[26]/46,XY[4]	cytoplasmic
NK 16	Blood	77	Yes	Yes	6	46,XY,t(6;9)(p23;q34),t(8;10)(q24;q11)[20]	nuclear
NK 17	Blood	84	No	Yes	9	46,XY[20]	cytoplasmic
NK 18	Blood	60	Yes	No		46,XX[20]	cytoplasmic
NK 19	BM	76	Yes	✞5m		46,XX[21]	cytoplasmic
NK 20	BM	80	Yes	Yes	6	46,XY[24]	cytoplasmic
NK 21	Blood	84	Yes	✞10m		46, XY [39]	cytoplasmic
NK 22	Blood	72	No	Yes	11	46,XX[28].	cytoplasmic
NK 23	BM	95	Yes	No		46,XY[29]	cytoplasmic
NK 24	Blood	63	Yes	✞11m		46,XX[20]	nuclear
NK 25	Blood	76	Yes	No		46,XX[20]	cytoplasmic
NK 26	BM	76	Yes	No		46,XY[26]	cytoplasmic

Supplementary Table 5: Clinical characteristics of the patients from GOELAMStheque with core-binding factor (CBF)-AML (n=24).

BM: bone marrow, CR: complete remission, MDS: myelodysplastic syndromes, ET: Essential Thrombocythaemia, NA : not available.

**Patient**	**BM or blood sample**	**% of blast cells**	**CR achieved with one course of treatment induction**	**Early disease relapse (<12months)**	**Time between diagnosis and relapse (month)**	**Caryotype**	**PCNA localization**
CBF 1	BM	78	Yes	No		46,XY,t(16;16)(p13;q22)[20]	cytoplasmic
CBF 2	BM	79	Yes	No		45,X,-Y,t(8;21)(q22;q22)[10]	nuclear
CBF 3	BM	81	Yes	No			cytoplasmic
CBF 4	BM	64	Yes	yes	11	46,XY,inv(16)(p13.1q22)[5]/46,idem,t(3;8)(q26;q21)[13]/46,XY[5]	cytoplasmic
CBF 5	Blood	88	Yes	No			cytoplasmic
CBF 6	Blood	76	Yes	No		46,XX,inv(16)(p13q22)[41]/48,sl,+11,+13 [2]/ 46,XX[11]	cytoplasmic
CBF 7	BM	79	Yes	Yes	7	46,XY,inv(16)(p13q22)[12]/46,XY[3]	nuclear
CBF 8	BM	86	Yes	No		46,XX,t(8;21)(q22;q22)[20]/46,XX[12]	cytoplasmic
CBF 9	BM	81	Yes	Yes	6	46,XX,inv(16)(p13q22)[7]/47,sl,+22[13]/47,sdl1,del(12q)[1]	cytoplasmic
CBF 10	Blood	75	Yes	Yes	12	46,XY,inv(16)(p13q22)[19]/46,XY[1]	cytoplasmic
CBF 11	BM	64	Yes	No		46,XY,t(8;21)(q22;q22)[12]/47,idem,+der(21)t(8;21)(q22;q22)[8]	cytoplasmic
CBF 12	BM	78	Yes	✞5m		47,XY,inv(16)(p13q22),+22[18]/48,idem,+8[2]	cytoplasmic
CBF 13	Blood	68	Yes	No		47,XY,inv(16)(p13q22),+22[9]/46,XY[3]	nuclear
CBF 14	BM	66	Yes	No		46, XY [2] / 47, XY, t(8;21) (q22;q22), +8, del (9) (q23q31) [39]	nuclear
CBF 15	Blood	72	Yes	Yes	4	45,X,-Y,t(8;21)(q22;q22)[17]/46,XY,t(8;21)(q22;q22),del(9)(q13q31)[3]	nuclear
CBF 16	BM	87	Yes	No		46,XY,t(21;8;3)(q22;q22;q21),ins(der(21)t(21;8;3);6)(21q22;6q11q27)[18]/46,XY[3]	cytoplasmic
CBF 17	BM	99	Yes	No		46,XX,t(16;16)(p13;q22)[?20]/46,XX[?3]	cytoplasmic
CBF 18	BM	86	Yes	No		46,XX,inv(16)(p13q22)[33]/46,XX[13]	nuclear
CBF 19	Blood	68	Yes	Yes	9	46, XX [1]/45,X,-X,t(8;21)(q22;q22) [19]	cytoplasmic
CBF 20	BM	82	Yes	Yes	11	46,XY,inv(16)(p13q22)[20]	nuclear
CBF 21	BM	89	Yes	Yes	2	45,X,-Y,t(8;21)(q22;q22)[21]	cytoplasmic
CBF 22	BM	67	Yes	Yes	11	46,XY,inv(16)(p13q22)[10]/48,idem,+9,+22[8]/46,XY[2]	nuclear
CBF 23	Blood	77	Yes	No		46,XY,inv(16)(p13q22)[12]/46,XY[8]	cytoplasmic
CBF 24	Blood	77	Yes	Yes	10	46,XY,del(7)(q21q35),inv(16)(p13q22)[20]	cytoplasmic
